# Photobiomodulation with Red and Near-Infrared Light Improves Viability and Modulates Expression of Mesenchymal and Apoptotic-Related Markers in Human Gingival Fibroblasts

**DOI:** 10.3390/ma14123427

**Published:** 2021-06-21

**Authors:** Ievgeniia Kocherova, Artur Bryja, Katarzyna Błochowiak, Mariusz Kaczmarek, Katarzyna Stefańska, Jacek Matys, Kinga Grzech-Leśniak, Marzena Dominiak, Paul Mozdziak, Bartosz Kempisty, Marta Dyszkiewicz-Konwińska

**Affiliations:** 1 Department of Anatomy, Poznan University of Medical Sciences, 60-781 Poznań, Poland; kocherova.evgenia@gmail.com (I.K.); abryja@ump.edu.pl (A.B.); bkempisty@ump.edu.pl (B.K.); 2 Department of Rheumatology, Center of Experimental Rheumatology, University Hospital Zurich, University of Zurich, 8952 Schlieren, Switzerland; 3 Department of Oral Surgery and Periodontology, Poznan University of Medical Sciences, 61-812 Poznań, Poland; kasia@naszdentysta.com.pl; 4 Department of Cancer Immunology, Chair of Medical Biotechnology, Poznan University of Medical Sciences, 61-866 Poznań, Poland; markacz@ump.edu.pl; 5 Gene Therapy Laboratory, Department of Cancer Diagnostics and Immunology, Greater Poland Cancer Centre, 61-866 Poznań, Poland; 6 Department of Histology and Embryology, Poznan University of Medical Sciences, 60-781 Poznań, Poland; k.stefanska94@o2.pl; 7 Laser Laboratory at Dental Surgery Department, Medical University of Wroclaw, 50-425 Wrocław, Poland; jacek.matys@wp.pl (J.M.); kg@periocare.pl (K.G.-L.); marzena.dominiak@umed.wroc.pl (M.D.); 8 Department of Periodontics, School of Dentistry Virginia Commonwealth University, VCU, Richmond, VA 23298, USA; 9 Physiology Graduate Program, North Carolina State University, Raleigh, NC 27695, USA; pemozdzi@ncsu.edu; 10 Prestage Department of Poultry Science, North Carolina State University, Raleigh, NC 27695, USA; 11 Department of Veterinary Surgery, Institute of Veterinary Medicine, Nicolaus Copernicus University in Torun, 87-100 Toruń, Poland; 12 Department of Biomaterials and Experimental Dentistry, Poznan University of Medical Sciences, 60-812 Poznań, Poland

**Keywords:** low-level laser treatment, LLLT, photobiomodulation, PBM, human gingival fibroblasts, in vitro

## Abstract

Photobiomodulation (PBM), also called low-level laser treatment (LLLT), has been considered a promising tool in periodontal treatment due to its anti-inflammatory and wound healing properties. However, photobiomodulation’s effectiveness depends on a combination of parameters, such as energy density, the duration and frequency of the irradiation sessions, and wavelength, which has been shown to play a key role in laser-tissue interaction. The objective of the study was to compare the in vitro effects of two different wavelengths—635 nm and 808 nm—on the human primary gingival fibroblasts in terms of viability, oxidative stress, inflammation markers, and specific gene expression during the four treatment sessions at power and energy density widely used in dental practice (100 mW, 4 J/cm^2^). PBM with both 635 and 808 nm at 4 J/cm^2^ increased the cell number, modulated extracellular oxidative stress and inflammation markers and decreased the susceptibility of human primary gingival fibroblasts to apoptosis through the downregulation of apoptotic-related genes (*P53*, *CASP9*, *BAX*). Moreover, modulation of mesenchymal markers expression (*CD90*, *CD105*) can reflect the possible changes in the differentiation status of irradiated fibroblasts. The most pronounced results were observed following the third irradiation session. They should be considered for the possible optimization of existing low-level laser irradiation protocols used in periodontal therapies.

## 1. Introduction

Since the first successful implementation of lasers in the mid-1960 for retina coagulation, lasers quickly have found their application in other fields of medicine, including dentistry [1]. In 1964, after high-energy lasers proved their effectiveness for bone ablation or osteotomy, Goldman et al. described the beneficial effects of the laser beam for dental caries treatment [2,3]. While high-energy lasers appeared to be a promising tool for hard tissue treatment, photobiomodulation (PBM), also called soft laser therapy or low-level laser irradiation (LLLI), may represent a treatment of choice for soft tissues. Low-level lasers cover a spectrum of red and near-infrared light (600–1100 nm), characterized by low absorption in water and the ability to penetrate biological tissues in a depth of 3 mm–15 mm [1,4].

Photobiomodulation has been used in periodontal treatment to stimulate repair and reduce pain and inflammation [5,6,7,8]. Application of PBM along with gingivectomy results in improved condition and faster regeneration [9]. In a study on wound healing after gingivectomy and gingivoplasty surgeries, low-level laser therapy application resulted in increased epithelialization and wound healing [10]. Laser irradiation was shown to be effective as an adjunctive treatment in promoting revascularization and pain control during the early healing of free gingival graft [10,11]. Moreover, LLLT was reported to exert a therapeutic effect in the nonsurgical treatment of chronic periodontitis [1,12,13]. Scaling and root planning combined with LLLT managed to improve radiographic bone density and to probe pocket depth short-term reduction in patients with chronic periodontitis [12].

The photobiomodulation effects on cells and periodontal tissues imply several complex mechanisms. Red and near-infrared light is primarily absorbed by cytochrome c oxidase in the respiratory chain of the mitochondria membrane. Following the cascade of reactions, cell signaling and messenger molecules are upregulated due to increased mitochondrial activity, including reactive oxygen species (ROS) and adenosine triphosphate (ATP) synthesis. The application of near-infrared light (810–1064 nm) stimulates light-sensitive ion channels increasing the levels of calcium ions (Ca^2+^) and its interaction with ROS and cyclic AMP (cAMP). All of these activities stimulate cell proliferation, migration and differentiation [7].

Additionally, near-infrared light has been suggested to increase the activity in the cell plasma membrane [14]. Low biochemical activity has been observed in wavelengths in the range of 700–770 nm. The optimum wavelength for near-infrared irradiation of biological tissues is usually considered to be around 810 nm [15]. However, some clinical trials have speculated that 630 to 660 nm may be the most effective wavelength to bring desirable effects on cells and tissues [16].

Notably, the effectiveness of photobiomodulation on the target tissues is dependent on a combination of parameters such as wavelength, energy density, the duration and frequency of the laser application [17]. The wavelength plays a key role in laser-tissue interaction, modulating the absorption and scattering characteristics [4,17]. Meanwhile, a biphasic dose-response affects the PBM clinical outcomes, indicating a therapeutic window for the optimal therapeutic reaction [18]. Despite the considerable efforts of accumulating in vitro and in vivo studies, the exact parameters remain controversial [12,19,20,21,22]. Substantial heterogeneity has been reported in the laser parameters and regimens among the different studies, with wavelengths ranging from 630 nm to 830 nm, output powers between 0.2 and 250 mW, and application frequencies ranging from 4 to 10 treatments [23]. The dose-dependent effects of PBM can be described by Arndt–Schultz’s curve, suggesting that the weak stimuli have the potential to enhance the physiological activity of treated cells and tissues. However in case of moderate stimuli, the activity is decreased, and extreme stimulation restrains and eliminates the activity [24,25] underlying the crucial role of the appropriate dosage for laser therapies. It has been revealed that the energy density ranging between 1–5 J/cm^2^ is optimal to achieve an optimal biological effect in different cells and organs, including periodontal tissues [23,24,26,27]. In addition to being used in various studies on different wavelengths, the doses falling within the range of 2–4 J/cm^2^ are mentioned in the World Association for Photobiomodulation Therapy (WALT) recommendations LLLT application in different fields of medicine [28].

The present study aimed to compare the in vitro effects of two different wavelengths. This included 635 nm and 808 nm on the human gingival fibroblasts in terms of viability, oxidative stress markers and specific genes expression during the four treatment sessions at power and energy density widely used in dental practice (100 mW, 4 J/cm^2^).

## 2. Materials and Methods

### 2.1. Cell Isolation

Human primary gingival fibroblasts were obtained from healthy gingival tissues of 12 patients (6 males, 6 females, age range 25–48 years old) undergoing impacted tooth extraction. The patients involved in the study had no systemic and metabolic diseases and did not receive any periodontal therapy within the last year. The patients with aggressive or generalized periodontitis, intraoral lesions, and smokers were excluded from the study. All patients meeting the criteria gave informed written consent to participate in the study.

Each gingival fragment was placed in a sterile 15 mL Falcon tube containing 5 mL of Dulbecco’s modified Eagle’s medium (DMEM, Sigma-Aldrich; Merck KGaA, Darmstadt, Germany). It was supplemented with 1% antibiotic/antimycotic solutions (Gibco; Thermo Fisher Scientific, Inc., Waltham, MA, USA) and transported to the laboratory for further cell isolation. Next, the samples were cut into small pieces and digested with collagenase type I (1 mg/mL) and Dispase II (1 mg/mL, both from Gibco; Thermo Fisher Scientific, Inc., Waltham, MA, USA) at 37 °C overnight.

**Figure 1:**
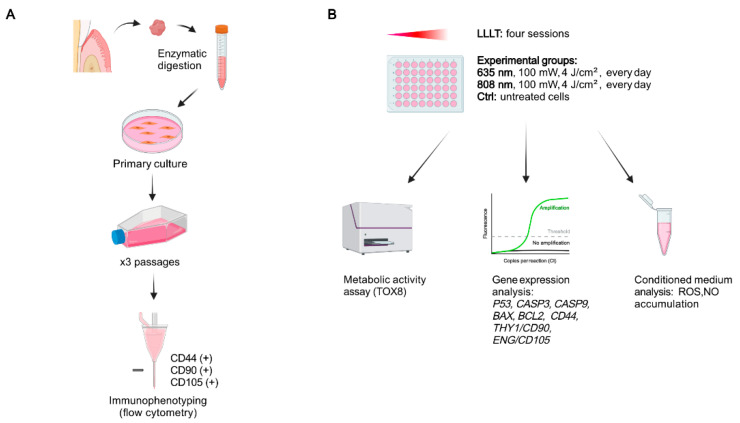
Schematic representation of the experimental design. (**A**) Gingival tissue samples were enzymatically digested to isolate the gingival fibroblasts for further primary in vitro culture. The cells were propagated and passaged three times and immunophenotyped before the experiment. (**B**) The cells were divided into control (untreated) and two experimental groups, which were subjected to LLLT sessions with red (635 nm) or near-infrared (808 nm) light. Following 24 h after each LLLT session, the metabolic activity assay was performed. The cells were harvested for gene expression analysis. The conditioned medium was collected to assess the level of extracellular oxidative stress and inflammation markers, created with BioRender.

The enzyme solution containing cells and tissue debris was centrifuged at 300× *g* for 8 min. The cellular pellet was resuspended in fresh DMEM supplemented with 10% fetal bovine serum (Sigma-Aldrich; Merck KGaA, Darmstadt, Germany), 4 mM L-glutamine (stock 200 mM; Gibco; Thermo Fisher Scientific, Inc., Waltham, MA, USA), 10 mg/mL gentamicin (Gibco; Thermo Fisher Scientific, Inc., Waltham, MA, USA), 10,000 U/mL penicillin and 10,000 µg/mL streptomycin (Gibco; Thermo Fisher Scientific, Inc., Waltham, MA, USA). Then it was transferred to T25 cell culture flasks and cultured at 37 °C in a humid 5% CO_2_ atmosphere. The culture medium was changed every 48 h until 80–90% confluence was reached. Cells were sub cultured using 0.25% trypsin-EDTA solution (Sigma-Aldrich; Merck KGaA, Darmstadt, Germany). The cells were propagated and passaged three times before the experiment (Figure 1).

### 2.2. Phenotypic Characterization

The mesenchymal character of isolated gingival fibroblasts was confirmed by investigating the presence of the following surface markers: *CD44*, *CD90*, and *CD105* with a FACScan flow cytometer (Becton Dickinson, San Jose, CA, USA). The following antibodies were used: *CD44*-PE (human, 130-113-897); *CD90*-FITC (human, clone: REA897, 130-114-901); *CD105*-APC (human, clone: REA794, 130-112-324) and REA Control (S)-PE (130-113-438), REA Control (S)-FITC (130-113-437), REA Control (S)-FITC (130-113-437), REA Control (S)-APC (130-113-434) and REA Control (S)-PE (130-113-438) from Miltenyi Biotec (Bergisch Gladbach, Germany). The data were analyzed using CellQuest Pro Software (Becton Dickinson, San Jose, CA, USA, version 5.2.1).

### 2.3. Laser Irradiation

To examine the effects of low-level laser irradiation, the gingival fibroblasts at the third passage were seeded into 48-well plates at the number of 5 × 10^3^ cells per well. Empty wells separated the experimental wells seeded with the cells to avoid overlapping of scattered irradiation. After 24 h, the culture medium was changed to remove the unattached cells. One group was treated with a diode laser emitting at the wavelength of 635 nm, power 100 mW and energy density 4 J/cm^2^. In contrast, another group was subjected to irradiation at 808 nm, 100 mW, 4 J/cm^2^, (handpiece diameter: 8 mm, spot area: 0.5024 cm^2^, average power density: 199.04 mW/cm^2^, continuous mode, dose: 4 J/cm^2^, time: 20 s, the total energy dose after all sessions were 16 J/cm^2^), (Smart M, Lasotronix, Poland). The cells were irradiated every 24 h during the four days. The untreated cells served as a control with medium changed every 24 h. Twenty four hours after each LLLI, we assessed the cell viability, selected genes expression, ROS and NO accumulation in the culture medium.

### 2.4. Cell Viability Assay

The number of viable cells in culture was estimated with TOX8 resazurin-based assay following the manufacturer’s protocol (TOX8, In vitro Toxicology Assay Kit, Sigma-Aldrich; Merck KGaA, Darmstadt, Germany). Briefly, following 24 h after each photobiomodulation, the culture medium was replaced with a fresh one containing 10% of the TOX8 dye solution. The plates were placed in the incubator for the next 4 h. Afterwards, 100 μL of medium from each well was transferred into a 96-well plate. The absorbance level was measured at 600 nm and 690 nm (reference wavelength) using a Synergy 2 plate reader (BioTek, Winooski, VT, USA). The decrease in absorbance is proportional to the metabolic activity and, consequently, to the number of living cells.

Furthermore, the number of viable cells was estimated based on the growth curve calculated in parallel with the cytotoxicity test. The cells were seeded at the density of 2.5 × 10^3^, 5 × 10^3^, 7.5 × 10^3^ and 10^4^ per well, and the level of dye absorbance was measured concerning the specific cells number to prepare the growth curve. The obtained linear trendline equation allowed us to estimate the number of cells.

### 2.5. Estimation of Extracellular Oxidative Stress and Inflammation Markers

The conditioned culture medium was collected from the wells following 24 h after each PBM treatment. ROS concentration in the conditioned medium was assessed by incubation with 2′,7′-dichlorodihydrofluorescein diacetate solution (H2D-CF-DA, Invitrogen, Thermo Fisher Scientific, Inc., Waltham, MA, USA) for 30 min at 37 °C and subsequent spectroscopic measurement at wavelengths of 495 nm and 529 nm. Nitric oxide (NO) concentration was determined with the Griess Reagent Kit (Invitrogen, Thermo Fisher Scientific, Inc., Waltham, MA, USA). Each samples’ absorbance was measured at 548 nm and converted to nitrite concentrations relative to the reference sample, following the manufacturer’s instructions. All procedures were performed in triplicate. The Synergy 2 (BioTek, Winooski, VT, USA) multi-mode plate reader was used for spectroscopic measurements.

### 2.6. Analysis of Chosen Genes Expression

Gingival fibroblasts were collected following 24 h after each PBM treatment. The process of RNA isolation was performed according to the modified Chomczyński and Sacchi method [29,30]. Briefly, the cells were suspended in a monophase solution of guanidine thiocyanate and phenol (TRI Reagent^®^, Sigma-Aldrich; Merck KGaA, Darmstadt, Germany). Next, the chloroform was added, with the samples centrifuged to obtain three separate phases. Total RNA located in the upper, aqueous phase was then precipitated with 2-propanol (Sigma-Aldrich; Merck KGaA, Darmstadt, Germany) and washed twice with 75% ethanol. Finally, RNA was dried and resuspended in 30 µL of pure water. RNA quantity and purity were examined spectrophotometrically (Epoch, Biotek, Bad Friedrichshall, Germany). Samples with a 260/280 absorbance coefficient greater than 1.8 were used for further experiments. The reverse transcription reaction was conducted according to the protocol provided by the manufacturer—SA Biosciences (RT2 First Stand kit-330401). RT-qPCR was performed using LightCycler (Roche Diagnostics GmbH, Mannheim, Germany). The amplification process was carried out using a 2 μL of cDNA solution, 18 μL of QuantiTect^®^ SYBR^®^ Green PCR (Master Mix Qiagen GmbH, Hilden, Germany) and primers (Table 1).

Relative gene expression was obtained using the 2^−ΔΔCt^ method [31]. The geometric mean of CT of β-actin (ACTB) and glyceraldehyde 3-phosphate dehydrogenase (GAPDH) was used as the reference.

### 2.7. Analysis of Chosen Genes Expression

Statistical analysis was carried out using GraphPad Prism 8 software (San Diego, CA, USA, version 8.0.1.244).

The Shapiro-Wilk test was used to verify the normality of the data distribution. One-way analysis of variance (ANOVA) with Tukey’s post hoc test was applied for multiple comparisons between the studied groups. The results are presented as mean ± standard deviation (SD), and *p* < 0.05 was considered statistically significant.

## 3. Results

**Figure 2:**
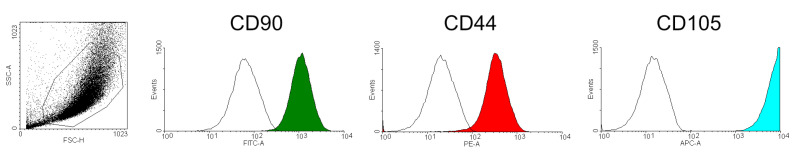
Phenotypical characterization of human primary gingival fibroblasts. Isolated cells were positive for *CD44*, *CD90*, and *CD105*.

The cells isolated from the gingival tissue samples presented the surface antigens typical for mesenchymal lineage cells, i.e., expression of *CD44*, *CD90*, and *CD105* markers (Figure 2).

**Figure 3:**
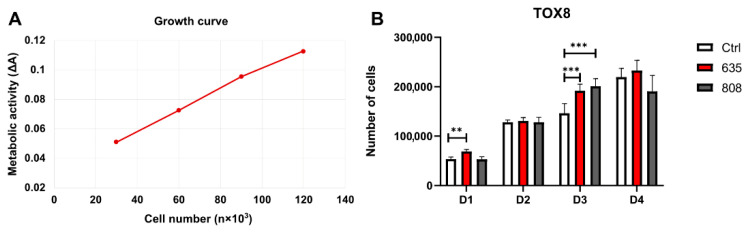
(**A**) The growth curve is showing the level of metabolic activity normalized to cell number. (**B**) Mean cells number in the irradiated and non-treated groups assessed with TOX8 metabolic activity assay. Results expressed as mean ± SD. ** *p* < 0.01, *** *p* < 0.001.

The resazurin-based assay (TOX8) revealed that photobiomodulation positively affected the cell number in gingival fibroblast cultures (Figure 3). The treatment with a 635 nm laser resulted in significant cell numbers 24 h after the first PBM (D1) compared to the control group. Instead, the second treatment (D2) did not influence the number of cells in the case of both 635 nm and 808 nm wavelengths. In contrast, the third PBM significantly improved this parameter in treated groups compared to control (D3). However, 24 h after the fourth PBM (D4), there was no significant difference between the irradiated and non-treated cells.

**Figure 4:**
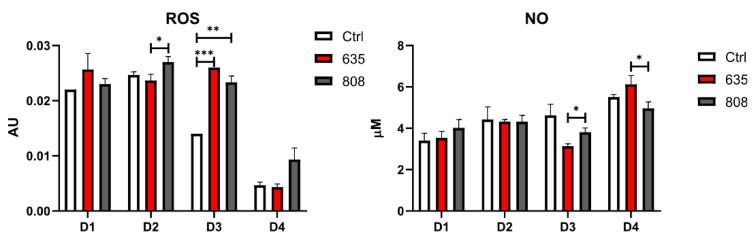
The levels of extracellular oxidative stress and inflammation markers in cultured human gingival fibroblasts. Results expressed as mean ± SD. * *p* < 0.05, ** *p* < 0.01, *** *p* < 0.001.

The conditioned medium was collected 24 h after every PBM treatment to assess the effects of photobiomodulation on the accumulation of molecular markers associated with oxidative stress and inflammation into the extracellular environment (Figure 4). After the second irradiation (D2), there was a significantly higher concentration of ROS in the medium collected from wells irradiated with 808 nm when compared to the 635 nm wavelength. Noticeably, the highest statistically significant differences were obtained after the third exposure to laser irradiation (D3). There was a significantly higher ROS accumulation in the conditioned medium of the gingival fibroblasts treated with 635 nm and 808 nm diode laser compared to the medium collected from the control group. After the fourth irradiation (D4), the differences between the experimental groups were not statistically significant. Interestingly, the ROS levels decreased considerably in all three groups when compared to earlier time points (D4).

No difference was noted in extracellular NO concentrations between the groups following the first two PBM treatments (D1, D2). The level of NO decreased significantly after the third exposure to 635 nm wavelength compared to 808 nm (D3). Interestingly, the fourth treatment revealed the opposite effect—NO concentrations rose significantly in wells exposed to 635 nm than 808 nm. Additionally, in contrast to ROS accumulation dynamics, there was a significant increase in NO concentrations following the fourth PBM (D4) compared to the third laser treatment (D3).

**Figure 5:**
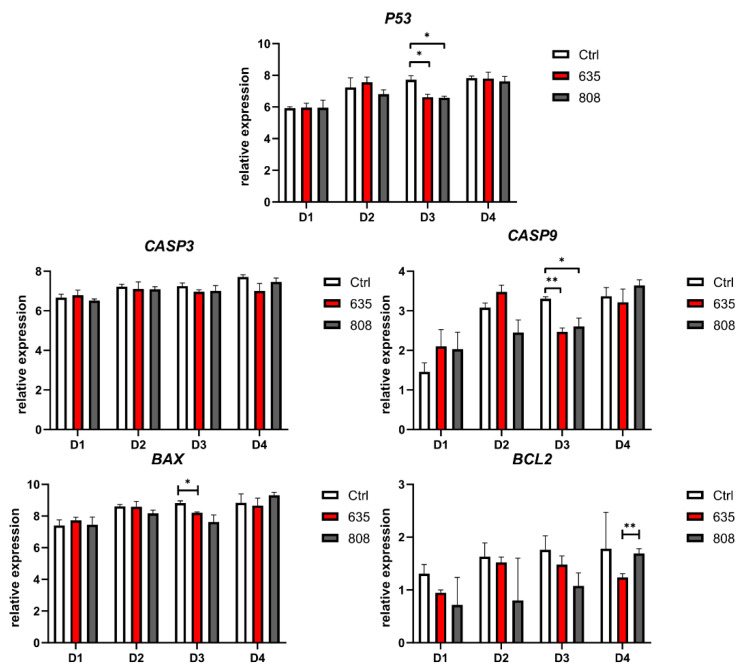
Expression of genes involved in apoptotic pathways in cultured human gingival fibroblasts. Results expressed as mean ± SD, n = 12. * *p* < 0.05, ** *p* < 0.01.

The RT-qPCR method demonstrated a significant decrease in pro-apoptotic *P53* and *CASP9* gene expression following the third (D3) exposure to 635 nm and 808 nm diode laser (Figure 5). The expression of *CASP3* remained unchanged in all groups. The downregulation of *BAX* was observed following the third exposure to 635 nm wavelength only (D3). After the fourth laser treatment, the expression of anti-apoptotic *BCL2* was significantly higher in cells subjected to the 808 nm wavelength compared to 635 nm.

**Figure 6:**
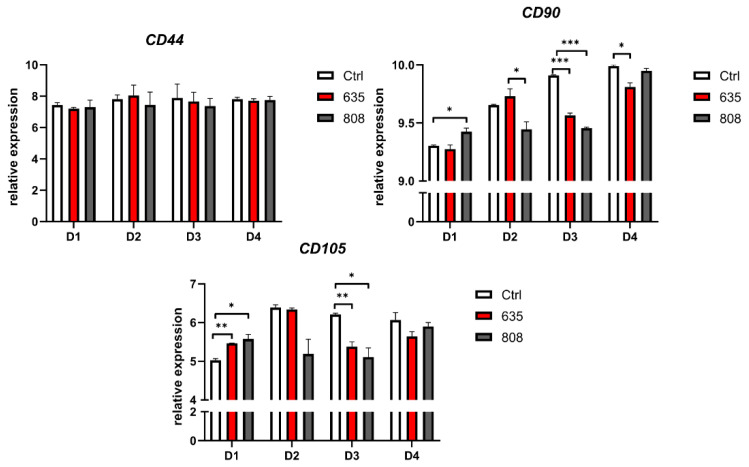
Gene expression of markers characteristic for mesenchymal stem cells in cultured human gingival fibroblasts. Results expressed as mean ± SD, n = 12. * *p* < 0.05, ** *p* < 0.01, *** *p* < 0.001.

RT-qPCR evaluation of *CD44*, *CD90*, and *CD105* gene expression demonstrated that PBM did not significantly affect the *CD44* mesenchymal gene expression (Figure 6). In contrast, the first stimulation with 808 nm upregulated the mesenchymal gene expression of both *CD90* and *CD105* genes (D1). Following the second PBM (D2), a significant *CD90* downregulation in the group exposed to 808 nm irradiation compared to the control. The observed decrease in *CD105* gene expression was not statistically significant. The third exposure resulted in significant *CD90* and *CD105* downregulation in the case of both wavelengths (D3). Following the fourth PBM with 635 nm, *CD90* expression remained significantly downregulated.

## 4. Discussion

The gingival tissue is composed of the superficial oral epithelium and underlying connective tissue. These tissues represent the first sites affected by interaction with biofilms and develop an inflammatory response. The gingival tissue is considered to be the initial location for destructive periodontal diseases [32].

As the main cells forming the connective tissue, fibroblasts are responsible for the extracellular matrix formation and maintaining the normal gingival tissue homeostasis. In the case of chronic pathological stimuli leading to connective tissue damage or managing loss of the gingival tissue, supplementing periodontal fibroblasts by grafting or stimulating cell migration, proliferation and viability seem to be a reasonable approach [33].

It has been documented that all these cell characteristics can be improved using photobiomodulation [34]. Almeida-Lopez et al. suggested enhanced human gingival fibroblasts proliferation in vitro following irradiation with diode laser at 670, 780, 692, or 782 nm and 2.0 J/cm^2^ [27]. It was noted that shorter exposure resulted in higher proliferation. Frozanfar et al. observed a significant increase in gingival fibroblasts (HGF3-PI53) proliferation on days 2 and 3 following the irradiation with 810 nm and 4.0 J/cm^2^ [35]. Additionally, day three was marked by the dramatically increased expression of the collagen type 1 gene in cultured cells. Periera et al. described 3- to 6-fold higher NIH-3T3 fibroblasts number following the treatment with 904 nm diode laser at 3.0 and 4.0 J/cm^2^ [36]. However, this effect was restricted to a small range of energy densities since the exposure to 5.0 J/cm^2^ had no significant effect on fibroblast growth.

Increased proliferation and improved viability in gingival fibroblasts were confirmed by deregulation of apoptosis-related gene expression [33,34,37,38,39,40,41,42]. The assessment of cell viability is important in studies on PBM effects. Photobiomodulation stimulates mitochondria by affecting their respiratory chain components, resulting in cAMP, ATP, and ROS production, directly influencing cell proliferation by initiating the intracellular signaling cascades [43]. The proliferation of cells may explain the steady growth of cell number between D1 and D3 in each group after the seeding. However, 635 nm wavelength seemed to induce the proliferation at the earlier time (D1) and stimulated its significant growth after the third irradiation session (D3). At D4, the difference between the irradiated and control cells was not significant, which underlines the importance of an appropriate dose and session number correction. PBM therapies should be adjusted to induce proliferation but avoid overstimulation which could have a cytotoxic effect because of excessive free radicals production [44]. The highest cell number was observed following the third application of both 635 and 808 nm PBM, corresponding to the significantly higher ROS accumulation in the culture medium at D3. By contrast, George et al. observed the lower ROS generated using 636 nm laser than the non-irradiated cells. The 825 nm laser, instead, provoked a significant increase in the level of ROS compared to the control [16]. Interestingly, the authors tested different laser irradiation parameters and concluded that the ROS production within biological systems is more dependent on the wavelength of the laser rather than energy density.

Treatment with 635 nm and 808 nm revealed similar effects in most assays. The physiologic and pathologic role of ROS in periodontitis has been studied for years. Studies suggest that although low ROS levels can be beneficial, excessive concentration of ROS can result in the initiation and exacerbation of periodontitis. There is still not enough data explaining crosstalk between ROS and autophagy in periodontal disease. However, data are suggesting that ROS may play a crucial role in determining cell fate by inducing autophagy or apoptosis [45].

Significant differences between the wavelength effects were observed in the case of extracellular NO concentration. The third irradiation session (D3) resulted in a significant decrease in NO level in the group treated with 635 nm, compared to 808 nm. In contrast, there was an increase in NO following the fourth treatment (D4). NO may represent one of the by-products of PBM mediating the cellular effects of the therapy [46]. Moreover, Karu et al. reported the irradiation-controlled mitochondrial NO signaling pathway in cultured cells [47]. In contrast, PBM has been shown to reduce inflammation, including NO synthesis [43,48,49,50]. However, the present data revealed no significant difference between the irradiated and non-treated cells.

In addition to cell viability, oxidative stress, and inflammation, the present study assessed the dynamics of mesenchymal marker expression in irradiated primary gingival fibroblasts. Several previous studies have shown fibroblasts share a mesenchymal stem cell phenotype, including the similar expression pattern of antigens characteristic for the mesenchymal stem cells (MSCs) and multilineage differentiation potential [51,52,53,54,55]. Surface immunophenotyping by flow cytometry confirmed *CD44*, *CD90*, *CD105* markers before the experiment. Next, the appropriate gene mRNA level was evaluated 24 h after each irradiation session to investigate the possible effect of PBM on mesenchymal-like features of the gingival fibroblasts. While the expression of *CD44* remained unchanged during the whole experiment, *CD90* and *CD105* were significantly modulated by laser treatment, with the most pronounced changes following the third exposure (D3). *CD90*, also known as Thy-1, is typically referred to as a mesenchymal marker associated with fibroblasts [56]. *CD90* was shown to control MSCs differentiation by acting as an obstacle in the pathway of differentiation commitment [57]. In fibroblasts, the existence of correlations between *CD90* expression and particular cell function was presumed. For example, in the study of Liu et al., fibroblasts revealed the heterogeneous *CD90* expression in lung fibroblasts [58]. Cells expressing *CD90* were more susceptible to apoptosis than cells lacking its expression. The authors showed that *CD90* expression is associated with decreased levels of anti-apoptotic molecules Bcl-2 and Bcl-xL and upregulation of cleaved caspase-9. The present study revealed that *CD90* downregulation at D3 was accompanied by the significant decrease in pro-apoptotic *BAX* expression following 635 nm laser irradiation suggesting a correlation between *CD90* and apoptosis-related factors expression.

Decreased *CD90* levels may reflect the differentiation of cultured fibroblasts. For example, Mokoena et al. reported that PBM at 660 nm with 5 J/cm^2^ successfully stimulated the human skin fibroblast differentiation into myofibroblasts [59]. Following 24, 48, and 72 h, the authors observed a significant increase in cell viability in the treated fibroblasts accompanied by decreased Thy-1 (*CD90*) expression and modulation of differentiation-related gene expression.

Interestingly, in the case of endoglin (*CD105*), the initial significant upregulation of gene expression followed the first treatment (D1). The third photobiomodulation resulted in the downregulation, similar to *CD90*. Endoglin, or *CD105*, is a type III coreceptor for TGF-β1, and its overexpression in fibroblasts was reported to affect physiological Smad/Alk1/Alk5 signaling to suppress the synthesis of TGF-β1 and extracellular matrix (ECM) proteins [60]. Endoglin may induce fibrosis development in different tissues, and its expression is higher in fibroblasts from fibrotic tissue than in non-fibrotic tissue [61]. *CD90* and *CD105* are overexpressed in carcinoma-associated fibroblasts [62]. PBM may modulate the differentiation status of cultured gingival fibroblasts, which agrees with recent studies describing that laser treatment may affect cell differentiation in human gingival fibroblasts, periodontal ligament cells, and stem cells [49,63,64]. The present study represents the first evidence of mesenchymal markers expression modulation in response to PBM in human primary gingival fibroblasts, which in part can explain the LLLI effects on cell differentiation reported in previous studies.

In conclusion, the present study revealed that PBM with 635 and 808 nm at 4 J/cm^2^ increased the cell proliferation, modulated extracellular oxidative stress and inflammation markers, and decreased the susceptibility of human primary gingival fibroblasts to apoptosis through the downregulation of apoptosis-related genes. Moreover, the data indicate that the modulation of *CD90* and *CD105* mesenchymal markers expression can reflect the possible changes in differentiation status of irradiated fibroblasts. The laser irradiation parameters used in the present in vitro study proved safe and exerted several beneficial effects on treated cells from healthy donors. However, further studies should consider the effectiveness of their application in the gingival tissues affected by pathological changes to elucidate the possible differences in biological response and, finally, to adjust the periodontal treatment protocols.

## Figures and Tables

**Table 1:** Sequences of primers used in RT-qPCR.

Gene	Primer Sequence (5′–3′)	Product Size (bp)
*P53*	GCTGAATGAGGCCTTGGAAC TTATGGCGGGAGGTAGACTG	114
*CASP3*	ATGTCGATGCAGCAAACCTC GCACACAAACAAAACTGCTCC	150
*CASP9*	TGATGTCGGTGCTCTTGAGA CGCAACTTCTCACAGTCGAT	162
*BAX*	TGACATGTTTTCTGACGGCA CACCCTGGTCTTGGATCCA	179
*BCL2*	ATGTGTGTGGAGAGCGTCAA GAAATCAAACAGAGGCCGCA	168
*CD44*	TCTGTGCAGCAAACAACACA TAGGGTTGCTGGGGTAGATG	234
*THY1/CD90*	CTAGTGGACCAGAGCCTTCG TGGAGTGCACACGTGTAGGT	236
*ENG/CD105*	CACTAGCCAGGTCTCGAAGG CTGAGGACCAGAAGCACCTC	165
*ACTB*	AAAGACCTGTACGCCAACAC CTCAGGAGGAGCAATGATCTTG	132
*GAPDH*	TCAGCCGCATCTTCTTTTGC ACGACCAAATCCGTTGACTC	90

## Data Availability

The data presented in this study are available on request from the corresponding author.

## Abbreviations

PBM	photobiomodulation
LLLI	low-level laser irradiation
LLLT	low-level laser treatment
ROS	reactive oxygen species
NO	nitric oxide
ATP	adenosine triphosphate
cAMP	cyclic adenosine monophosphate
H2D-CF-DA	2′,7′-dichlorodihydrofluorescein diacetate
DMEM	Dulbecco’s modified Eagle’s medium
MSCs	mesenchymal stem cells
ECM	extracellular matrix
WALT	World Association for Photobiomodulation Therapy
ACTB	β-actin
GAPDH	glyceraldehyde 3-phosphate dehydrogenase
P53	tumor protein P53
CASP3	caspase 3
CASP9	caspase 9
BAX	BCL2 associated X protein
BCL2	B-cell lymphoma 2
CD44	cluster of differentiation 44
THY1/CD90	thymocyte differentiation antigen 1/cluster of differentiation 90
ENG/CD105	endoglin/cluster of differentiation 105
AU	arbitrary units
